# Supplementary material for: Structure and Long-Term Stability of the Microbiome in Diverse Diatom Cultures
Source: Microbiol Spectr. 2021 Jun 30;9(1):10.1128/spectrum.00269-21. doi: 10.1128/spectrum.00269-21 (PMC8552671; doi:10.1128/spectrum.00269-21)
Supplement: SUPPLEMENTAL FILE 1 — Download SPECTRUM00269-21_Supp_1_seq8.pdf, PDF file, 1.2 MB [file spectrum00269-21_supp_1_seq8.pdf]

Supplemental Table S1. Detailed collection site data. Spreadsheet file MorrisAshworth.TableS1.csv

Table S2. Most abundant OTU in each sample. * indicates the genus was identified as the best match in a blastn search. # samples indicates the total number of samples in which the OTU was detected.

Sample	OTU	Phylum/Class	Genus	Abundance	Overall Abundance	# Samples
1.1	OTU2	Alphaproteobacteria	Unclassified	48.20%	7.20%	5
1.2	OTU2	Alphaproteobacteria	Unclassified	37.70%	7.20%	5
1.3	OTU2	Alphaproteobacteria	Unclassified	22.00%	7.20%	5
1.4	OTU44	Bacteroidetes	Unclassified	13.70%	1.06%	5
2.1	OTU1	Alphaproteobacteria	*Hyphomonas*	36.90%	8.62%	14
2.2	OTU4	Alphaproteobacteria	*Oceanicola*	59.40%	4.47%	13
3.1	OTU34	Bacteroidetes	*Ekhidna*	52.40%	3.83%	14
3.2	OTU17	Planctomycetes	*Phycisphaera*	22.80%	1.52%	1
4	OTU3	Alphaproteobacteria	Sphingomonadales	50.30%	3.65%	8
5	OTU7	Bacteria unclassified	*Leptospira**	19.40%	2.21%	2
6	OTU13	Gammaproteobacteria	*Alcanivorax*	18.60%	1.55%	5
7	OTU11	Alphaproteobacteria	Unclassified	14.70%	1.86%	6
8	OTU1	Alphaproteobacteria	*Hyphomonas*	51.50%	8.62%	14
9	OTU12	Gammaproteobacteria	Unclassified	33.60%	2.63%	4
10	OTU5	Gammaproteobacteria	Unclassified	26.60%	4.01%	4

Table S3. OTUs shared amongst cultures from different sites.

	Gab Gab 2008	Gab Gab 2011	Achang	Agat
Gab Gab 2008	26	14	12	8
Gab Gab 2011		73	14	10
Achang			29	10
Agat				22

Table S4. OTUs shared amongst cultures of different species.

	Astrosyne	Roundia	Florella
Astrosyne	9	6	4
Roundia		29	10
Florella			22

Table S5. P-values from AMOVA tests.

Grouping[1]	Theta-YC	Bray-Curtis	Jaccard Traditional	Jaccard Abundance
Sample Locale[2]	n.s.[7]	n.s.	0.077	n.s.
Sample Site/Time[3]	0.001	0.001	0.001	0.008
Diatom Order[4]	0.074	0.058	0.075	n.s.
Diatom Species[5]	n.s.	n.s.	0.021	n.s.
Diatom Culture[6]	0.014	0.014	0.005	0.032

1 Each AMOVA analysis was performed using four different distance metrics on a subset of the full dataset such that groupings with at least two observations per group were compared.
2 Sample Locale grouping compared cultures initially obtained from Guam vs. the Gulf of Mexico.
3 Sample Site/Time grouping compared cultures collected at the same site and time to culture collected at different sites and times. The groups considered where Gab Gab 2008, Gab Gab 2011, Achang, and Agat.
4 Samples from cultures of diatoms from the same taxonomic order were compared: Cyclophorales vs. Striatellales.
5 Samples from cultures of diatoms of the same species were compared: *Astrosyne radiata*, *Roundia cardiophora*, and *Florella pascuensis*
6 Multiple samples from the same culture (Table 1) were compared: Culture 1, Culture 2, and Culture 3.

Table S6. AMOVA Results. Spreadsheet file MorrisAshworth.TableS6.csv

Table S7. P-values of post-hoc pairwise comparisons from significant AMOVA tests.

Pairwise Comparison	Theta-YC	Bray-Curtis	Jaccard Traditional	Jaccard Abundance	Average
**Sample Site/Time:**					
Achang – Agat	0.677	0.333	0.355	1.000	0.591
Achang – Gab Gab 2008	0.067	0.108	0.080	0.055	0.078
Achang – Gab Gab 2011	0.341	0.333	0.341	0.341	0.339
Agat – Gab Gab 2008	0.135	0.067	0.069	0.182	0.113
Agat – Gab Gab 2011	0.353	0.333	0.353	0.665	0.426
Gab Gab 2008 – Gab Gab 2011	0.075	0.041	0.075	0.044	0.059
**Diatom Culture:**					
Culture 1 – Culture 2	0.050	0.108	0.067	0.044	0.067
Culture 1 – Culture 3	0.133	0.067	0.066	0.178	0.111
Culture 2 – Culture 3	0.666	0.333	0.333	1.000	0.583

Table S8. OTUs with significantly different abundances in cultures collected from Gab Gab Beach in 2008 vs. 2011. Abundances are counts per thousand and represent means from all observed samples of cultures from the two sites. *, OTU is present in more than half of all examined cultures. P-values were determined from Metastats analysis. Only hits from the first 100 OTUs are shown.

OTU	Taxonomy	2008	2011	p-value
OTU2	Alphaproteobacteria unclassified	269.8	0.09	0.021
OTU11	Alphaproteobacteria unclassified	1.4	131.7	0.005
OTU18*	Rhodobacteraceae unclassified	26.3	0	0.032
OTU19*	Gammaproteobacteria unclassified	11.2	0	0.042
OTU22	Rhodobacteraceae unclassified	0.91	0	0.042
OTU56	Bacteroidetes unclassified	0	21.2	0.013
OTU57	*Balneola*	9.8	0.91	0.032
OTU70	Saprospiraceae unclassified	0	14.1	0.005
OTU86	Alphaproteobacteria unclassified	0.63	0	0.017

Table S9. OTUs with significantly different abundances in cultures collected from Gab Gab Beach in 2008 vs. Achang Reef. Abundances are counts per thousand and represent means from all observed samples of cultures from the two sites. P-values were determined from Metastats analysis. Only hits from the first 100 OTUs are shown.

OTU	Taxonomy	Achang	Gab Gab 2008	p-value
OTU2	Alphaproteobacteria unclassified	0	269.8	0.027
OTU57	*Balneola*	0	9.8	0.039
OTU86	Alphaproteobacteria unclassified	8.1	0.63	0.020

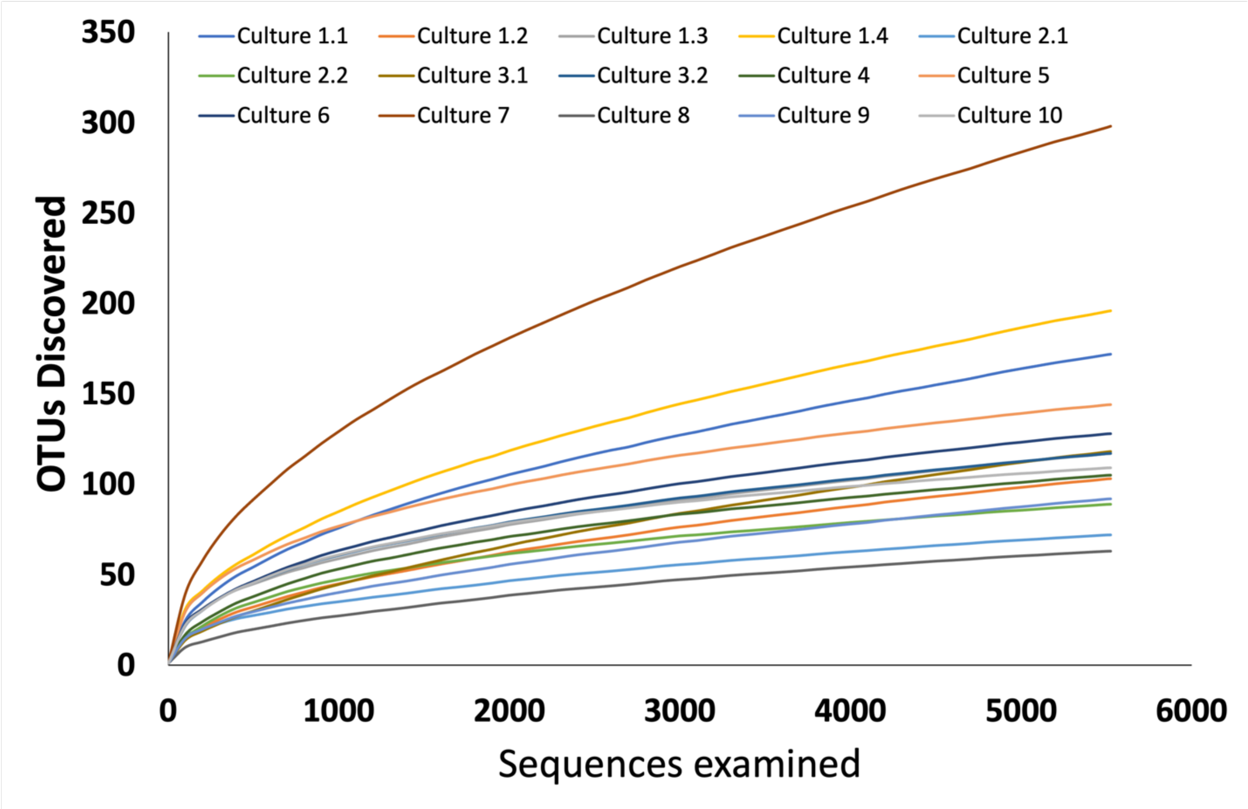

Figure S1. Rarefaction analysis of sequencing. Culture designations correspond to the names given in Table S1.

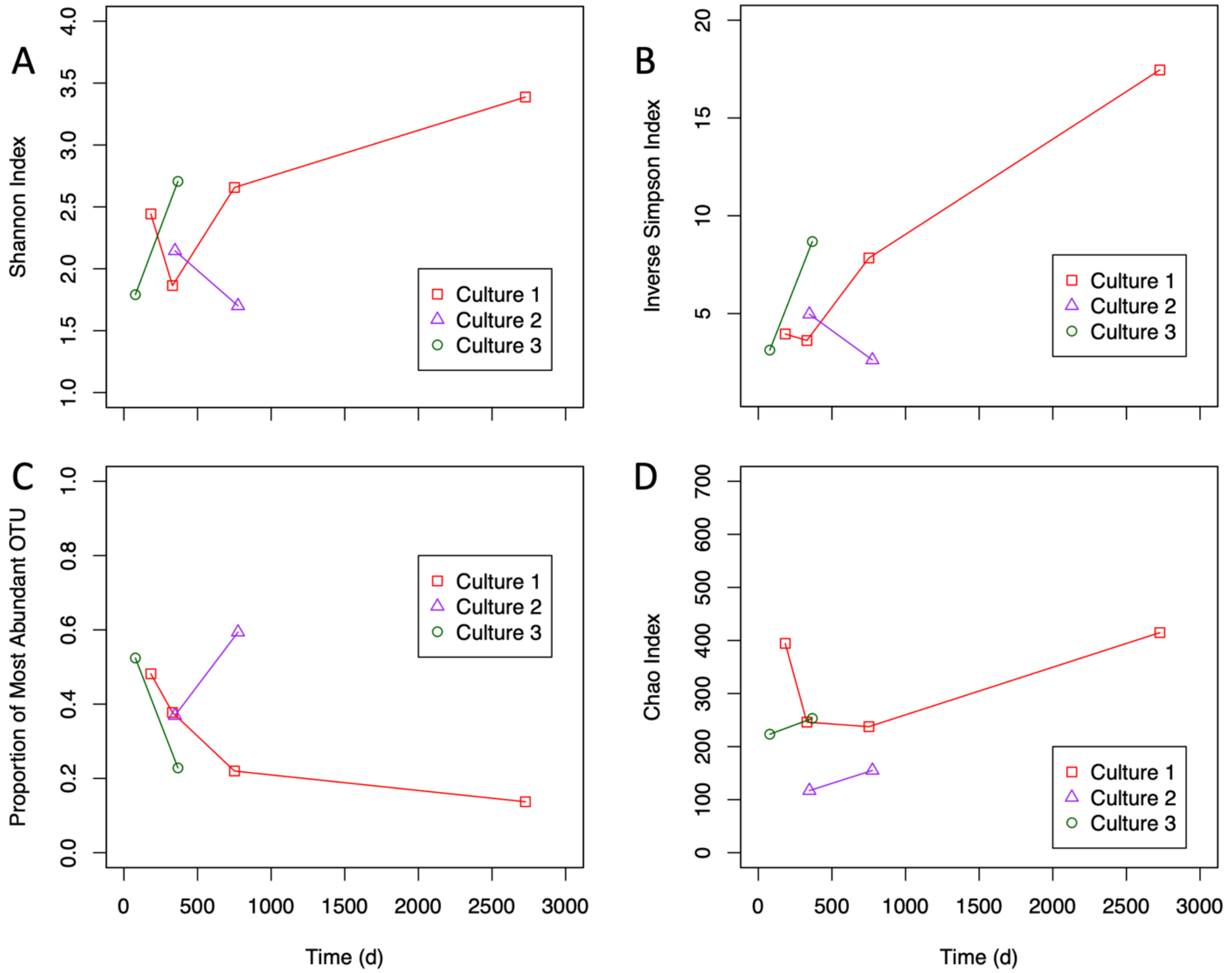

Figure S2. Changes in culture diversity metrics over time in multiply sampled cultures. Each line connects successive time-point samples from the same diatom culture. Culture 1, *Astrosyne radiata*; Culture 2, *Roundia cardiophora*; Culture 3, *Florella pascuensis*.

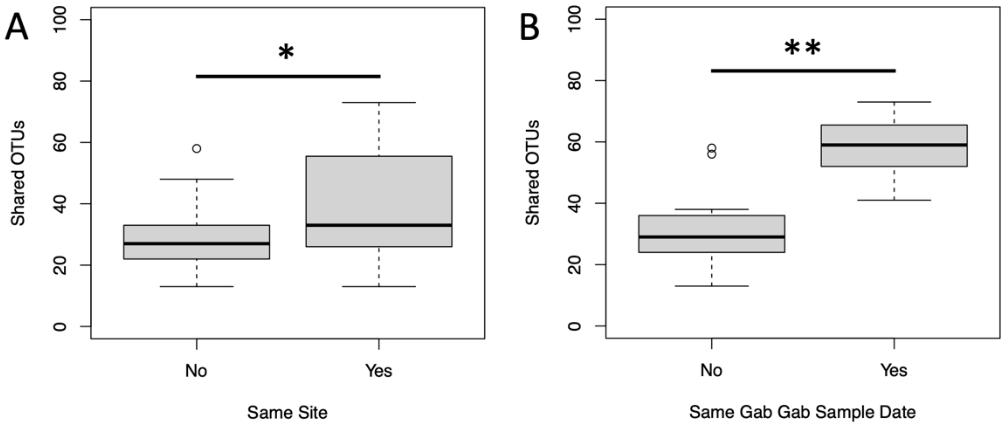

Figure S3. Shared OTUs by Site and Sampling Time. A. Significantly more OTUs were shared between samples of cultures collected at the same site than different sites. B. For samples collected at the same site, repeated samplings of cultures initially collected on the same day had more shared OTUs with each other than with samplings from other dates. Asterisks represent the result of Mann-Whitney U tests comparing the two groups shown; *, $p < 0.05$; **, $p < 0.01$.

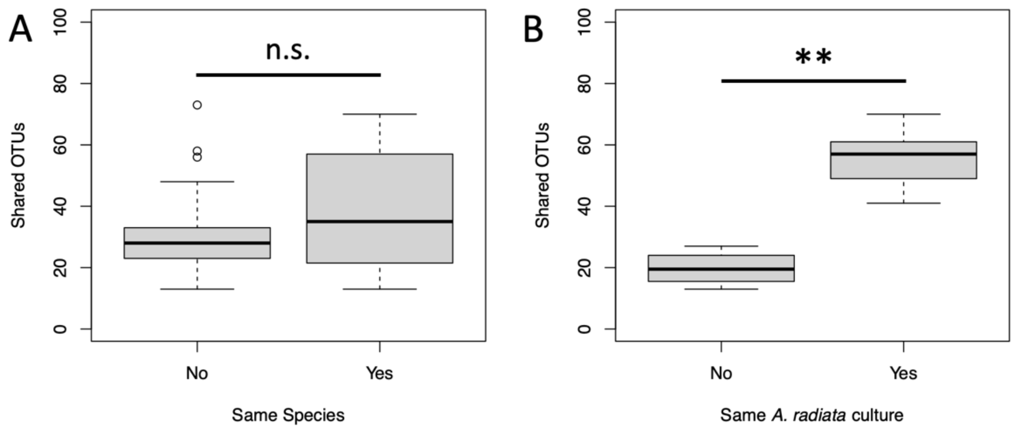

Figure S4. Shared OTUs by Species. A. The number of shared OTUs between cultures of the same diatom species was not statistically greater than the number of shared OTUs between cultures of different diatoms. B. Repeated samples from one *Astrosyne radiata* culture shared more OTUs with each other than any of them did with an *A. radiata* culture collected at the same site but at a different time. Asterisks represent the result of Mann-Whitney U tests comparing the two groups shown; n.s., not significant ($p > 0.05$); **, $p < 0.01$.

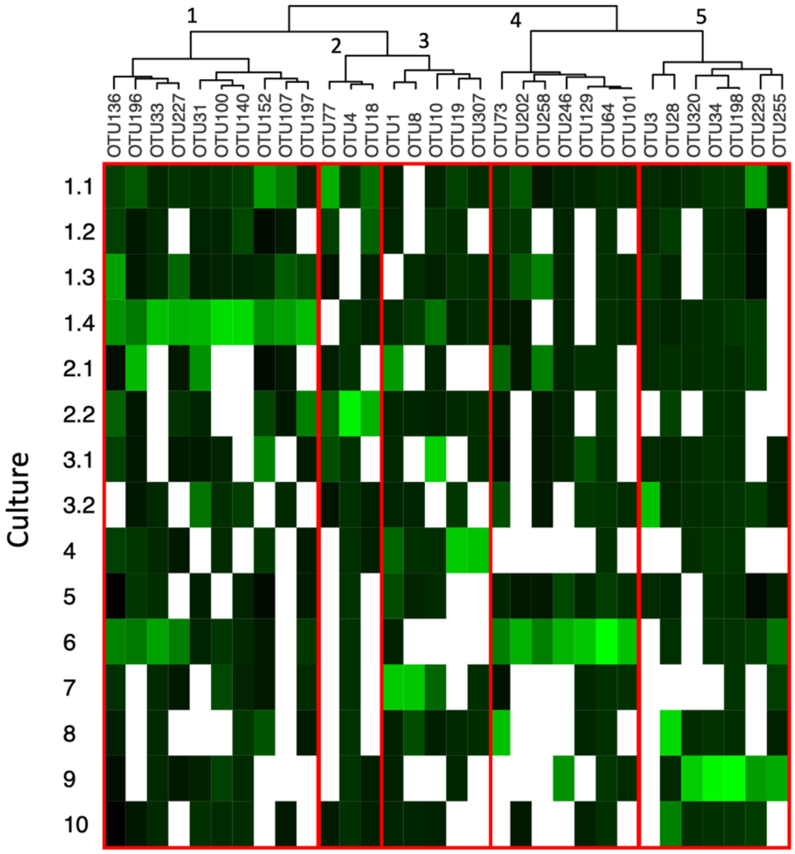

Figure S5. Abundance of ubiquitous OTUs across samples. White boxes indicate the OTU was not detected in the sample. Colors ranging from black (low) to green (high) indicate relative abundance in each sample, scaled for each OTU to a mean of 0 and a standard deviation of 1. Dendrogram and node numbering are derived from the hierarchical clustering algorithm described in main text Figure 3; red boxes enclose the five clusters.

Figure S6. Diatom microbiome community structure. This animated NMDS plot shows how the various cultures cluster together in three dimensional space. Arrows connecting spheres show multiply sampled cultures, going from earlier to later samples. The light-gray arrows are vectors representing the influence the most impactful OTUs on community position in the plot; the size of the arrow and arrowhead scale with the ubiquity of the OTU. Only OTU1 was found in >50% of samples. (Note: movie file available as separate download)
