# Supplementary figures and images for: Structure and Long-Term Stability of the Microbiome in Diverse Diatom Cultures
Source: Microbiol Spectr. 2021 Jun 30;9(1):10.1128/spectrum.00269-21. doi: 10.1128/spectrum.00269-21 (PMC8552671; doi:10.1128/spectrum.00269-21)

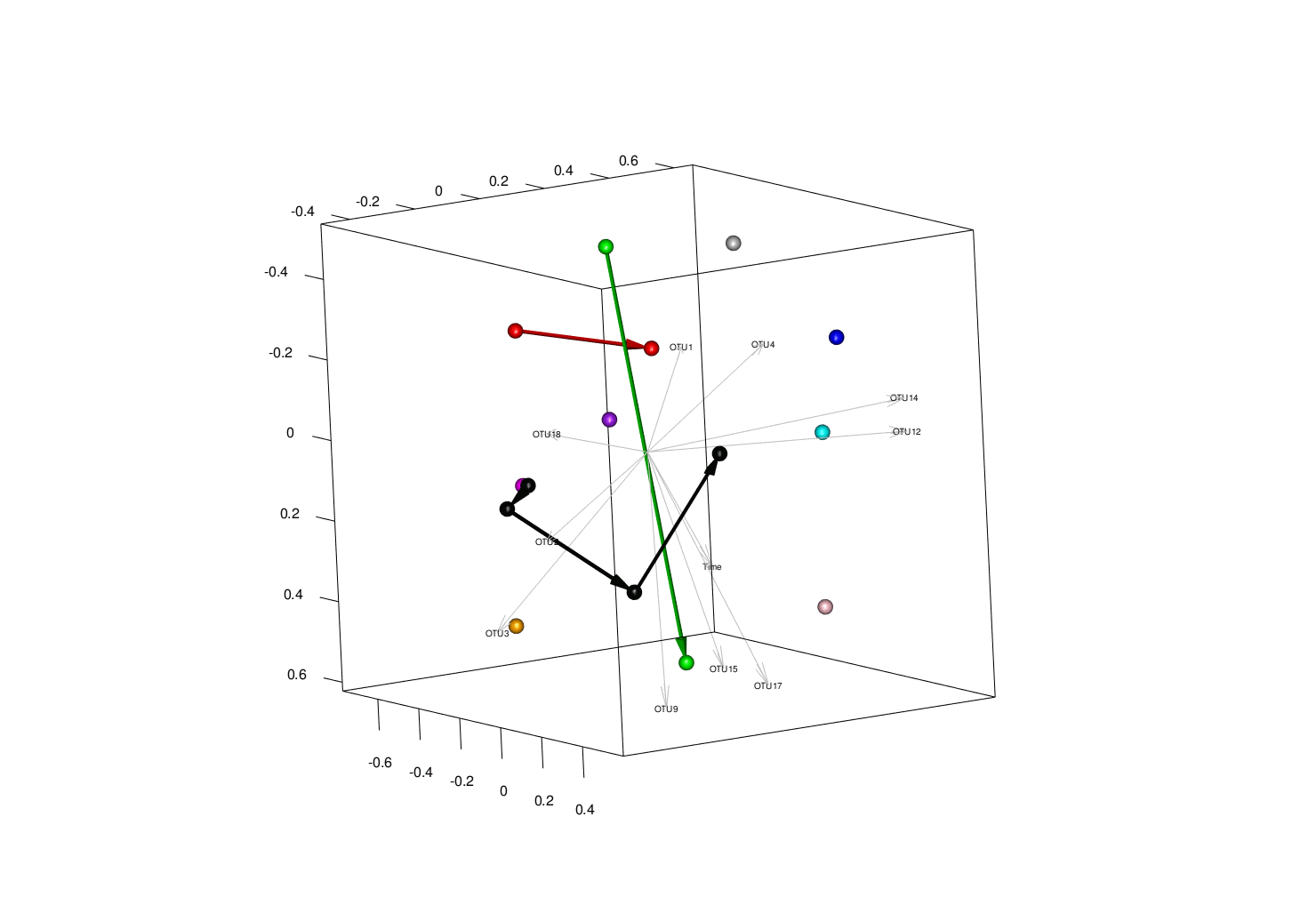

Supplement: SUPPLEMENTAL FILE 4 — Download SPECTRUM00269-21_Supp_4_seq11.gif, GIF file, 20.3 MB [file spectrum00269-21_supp_4_seq11.gif]
